# How useful are child death reviews: a local area’s perspective

**DOI:** 10.1186/1756-0500-6-295

**Published:** 2013-07-26

**Authors:** Francesca Mazzola, Abdu Mohiddin, Malcolm Ward, Gillian Holdsworth

**Affiliations:** 1 Public Health Directorate, NHS Lambeth, 1 Lower Marsh, London, UK; 2 Social Care, Southwark Council, 160 Tooley Street, London SE1P 5LX UK; 3 Southwark BSU, 160 Tooley Street, London SE1P 5LX, UK

**Keywords:** Child death review, Overview panel, Modifiable

## Abstract

**Background:**

Child Death Overview Panels (CDOP) provide a multidisciplinary and confidential forum to learn from and reduce deaths in those under 18 years. How well they perform and how to improve their effectiveness is a question posed at both local and national levels in England. With this in mind, this study looked at the child death review process in two London boroughs with a joint CDOP.

**Findings:**

Data on cases reviewed from April 2008 to January 2011 were analysed focusing on cause of death and modifiable factors. Key stakeholders involved in the child death review process were interviewed regarding the effectiveness of the local death review process with responses analysed thematically.

105 (50.5%) of all notified deaths were reviewed to completion by CDOP of which 26.7% had modifiable factors. Neonates were the largest group of deaths (42.8%). Stakeholders found reviews time consuming, required significant administration and better integration with related processes e.g. hospital mortality meetings. Too much time was spent analysing cases of limited modifiability such as neonates. Implementation of recommendations needed strengthening and inclusion into the wider health and social care economy including joint strategic needs assessments and commissioning processes. Delayed reporting of information on cases contributed to a backlog.

**Conclusions:**

The current process is bureaucratic, should better address neonatal deaths and needs more focus on implementing recommendations. Solutions include simpler forms, neonates-only subgroups, and linking recommendations to strategic initiatives such as Health and Wellbeing Boards.

## Findings

### Background

In April 2008 Child Death Overview Panels (CDOPs) became mandatory in England with every local authority required to have a CDOP and to produce an annual report for its Local Safeguarding Children Board (LSCB) [1].Child death review systems have been in place in some countries, notably Australia and the USA for many years and were brought about by concerns over deaths due to maltreatment [2,3].

A key function of CDOP is to identify if a child’s death was “modifiable” i.e. did factors exist which may have contributed to the death and which, by means of nationally or locally achievable interventions, could have been modified to reduce the risk of future child deaths. An annual return is made to the national government on the numbers of both child deaths and those deemed modifiable. The child death review has two elements: a rapid response to unexpected deaths by a group of involved professionals who enquire into and evaluate such deaths; and, an independent and analytic overview of all child deaths up to the age of 18 years of age (excluding stillbirths) which happens at a later stage as part of a multidisciplinary panel discussion – the CDOP. CDOP is a confidential review in which representatives of the services involved discuss cases without any parents/carers or family of the deceased present.

A review of “early starter” CDOPs made recommendations to inform all panels including the need for a clear remit and purpose, robust structures and processes, committed personnel and a multiagency approach [4].However, concerns have been raised regarding how well child death reviews work in practice both in England and elsewhere. Experience from the USA suggests that panels can have a misplaced focus on ‘assessing the problem’ that may have contributed to a death rather than ‘proposing solutions and implementing them [5].Further, a review of US states’ child death programmes found variation between states which adversely affected prevention including at the national level [6]. In England, an independent review of child protection commissioned by the Government acknowledged evidence of some good local learning from CDOP, but identified the lack of a national mechanism for analysing, collating, and disseminating local learning [7].

We sought to review our local CDOP which covered two London boroughs and meets approximately monthly with a chair from Social Services. The review was initiated by the two LSCBs to ascertain CDOP’s effectiveness and identify areas for improvement given its unchanged operation since 2008. Our local CDOP operation is informed by the London and national requirements and is a busy one, with about 70 deaths reviewed annually (compared to 36 per London CDOP and 42 for England) [8].

Both boroughs are ethnically diverse, deprived and have high rates of child death (infant mortality and 1–18 years mortality are higher than seen for London and nationally) [9].

## Methods

### Child death data

The child review process uses nationally set forms and data was extracted from these from 1st April 2008 to 31st January 2011: Form Bs (agency report form for cases), Form C (analysis proforma summarising the Panel’s findings) and postmortem reports where available. Form Cs include information on whether modifiable features are considered to play a part in a child’s death.

### Stakeholder consultation

Semi-structured interviews were conducted with key stakeholders, both those who attend CDOP meetings and others who do not but are part of the overall child death review process. Stakeholders were paediatricians (acute and community), midwives, neonatologists, social care professionals, public health specialists, administrative staff, members of the safeguarding team and named child protection and safeguarding nurses. The interviews elicited views on the process as a whole and had follow-up questions on its main components such as the rapid response to a death, administration/information gathering, the Panel itself, identifying modifiability and implementation of findings. Bereavement counsellors were consulted both in their own roles and also as representatives of parents/carers who were not consulted given the confidential nature of CDOP.

The responses were recorded electronically and analysed using the thematic framework approach.

Ethical approval was not required for this study as it was an evaluation of an existing service (as per the research governance framework of the NHS Health Research Authority).

## Results

### Child death review activity

208 cases were notified from 1st April 2008 – 31st January 2011, of which 105 (50.5%) were reviewed to completion by CDOP. 61 (29.3%) of the 208 cases notified underwent a rapid response meeting.

Of the 105 cases completely reviewed, 65 (61.9%) were completed within 12 months of notification, with the longest taking 22 months. 78 (74.2%) of child deaths occurred in children less than one year of age. 55 (52%) of deaths were male, with the remainder female. The commonest ethnicities amongst the child deaths were Black African (28, 26.7%) and White British (15, 14.3%).

### Cause of death and modifiable features

The main causes of death in the 105 reviewed were perinatal and neonatal events (n = 45, 42.8%), followed by chromosomal, genetic and congenital abnormalities (n = 19, 18.1%), then acute medical or surgical conditions (n = 10, 9.5%).

The deaths were also categorised into groups determined at national level: 45 (42.8%) were neonatal deaths, 22 (20.9%) were unexpected deaths and the remainder grouped as either SUDI or ‘expected’ (excluding neonatal deaths).

No modifiable factors were identified in 69 (65.7%) cases, and modifiable factors were identified in 28 (26.7%). 8 (7.6%) cases were deemed to have either inadequate or no information to reach a decision regarding modifiability. Unexpected deaths were the most modifiable (54.5%) and expected deaths the least (5.3%).

### The local CDOP operation

This follows national (and London) guidance and met all the requirements such as quoracy, membership, attendance, funding (as per the national grant) though the need for a chair from an independent service was not (hence the move to a chair from public health). Case discussions were led by the Chair who invited members to contribute where relevant and a general discussion then ensued resulting in the completion of the form C.

The CDOP chair was accountable to the LSCB Chair and a report from the Panel was regularly required by both the LSCB's (at least biannually).

### Stakeholder interviews

25 stakeholders were approached and 18 were interviewed from a broad range of services with the key themes described below. Table 1 has a summary of the themes with illustrative quotes.

**Table 1:** Summary of key themes raised in stakeholder interviews

**Theme**	**Typical response**
**Information gathering**	
Problems with form B*: gaps in its completion, needs to be simpler	‘Duplication on Form Bs from different agencies’, ‘only new information should be added by each agency’
Timeline for form B completion issued	‘A national time span for Form B completion should be implemented’
Better liaison with the coroner regarding post-mortem information	‘Representative of coroner should be present at CDOP’
Incorporate rapid response, Serious Case Review meetings, Serious Incident (SI), Mortality and Morbidity (MM) hospital findings better into information gathering system	‘Parallel processes that don’t necessarily converge at CDOP’
Better approach to neonatal deaths, especially eliciting additional maternal information	‘Shift in services to focus more on neonatal and maternal factors’,
**CDOP Meeting**	
Quality assure forms prior to the CDOP meeting	“Improve the quality of information for panel members to review prior to CDOP discussion”
Information shared with members prior to Panel meeting	‘Seeing the information beforehand would streamline the CDOP meetings, less questions would be asked’
Triage system for some cases that do not need to come to CDOP with particular concern about extremely premature deaths	‘Separate under 1 years old’ ‘focus on multi-agency cases’, ‘screen out expected deaths’
Less delay in completing cases	‘Delay in CDOP completion of case risks losing the meaning of the case’
Terminology used is difficult to interpret	‘Categorisation as expected and unexpected differs between different professions’, ‘huge discrepancy between what is termed modifiable and non-modifiable’
More public health specialist involvement and leadership	‘Health lead instead of safeguarding lead’
**Lessons learnt**	
More required to implement lessons learnt	‘Unclear of how lessons learnt are followed up’, ‘feedback given but not in an auditable fashion’
Regular update seminars on lessons learnt	‘Regular update seminars with designated people from each agency’
Better sharing of lessons learnt between CDOPs nationally	‘Need to pull information together’, ‘feel like we are working in isolation’
**Process as a whole**	
Death review process is still developmental	‘A work in progress’
Good multi-agency review of child deaths	‘Good multi-agency review’, ‘the only multi-agency review’, ‘allows access to all information’, ‘encourages to think outside the box’
Greater commitment and awareness of Child Death Review Process needed	‘Feels like an add-on’, ‘often not a high-priority’, ‘more ownership from agencies’
Time and resource consuming	‘Labour intensive’, ‘big time commitment for already busy people’

Key: ^*^ form B – the nationally prescribed data collection form for cases, sent out to all relevant agencies to complete by CDOP administration.

The main findings can be divided into four distinct sections; i) issues with the CDOP meetings themselves, ii) issues surrounding the implementation of lessons learnt, iii) the logistical obstacles of information gathering and iv) the child death review process as a whole.

The problems identified in the CDOP meetings were both logistical, for example regarding information sharing prior to the meeting and long delays in completion of cases, and ideological. There was a lack of clarity regarding the terminology employed by the Child Death Review process, with much of the meetings discerning whether the death was to be classified as modifiable or non-modifiable rather than on lessons to be learnt from the case. This was partly related to national guidance to change the original term used “preventability” to “modifiability”. It was felt that the types of deaths attributed to each category varied between meetings, and a similar issue was found for classifying expected and unexpected deaths. This was especially true for cases in intensive care, where significant morbidity and mortality can be expected, compared to cases in the community.

Implementation of lessons learned and identifying measurable outcomes of these changes were issues raised. This highlights the challenge the CDOP faces in achieving its main final aim: the assessment and prevention of child deaths. In the current system, there is no clear guidance on whose responsibility it is to decide a functional and realistic plan, who is in charge of its implementation and methods for determining the success of the recommendation. Central guidance on prioritisation of recommendations would also be of assistance, although local epidemiology is likely to influence the urgency for each specific area. This was highlighted as the most frustrating aspect of the CDOP, as hard work was not readily translated into helpful action.

On a more practical level, it was reported that the CDOP meetings themselves had significant scope for improvement. Shortage of staff and funding were evident, but many stakeholders identified areas in which they thought the running of the meeting and the flow of cases could be improved:

•Sharing of background information with the CDOP meeting members sufficiently prior to the meeting so they can familiarise themselves with the material.

•Less delay in processing cases so that lessons learnt from them can be quickly identified and applied.

•Triaging cases that are unlikely to yield new information on child deaths, such as premature neonatal deaths and expected deaths.

•Public health chairing CDOP meetings to bring in wider population issues instead of individual cases as such cases are often covered by other agencies.

•Using simpler data collection tools (e.g. form A and C alone) would reduce the work as form B completion is often a rate limiting step; also, the introduction of target deadlines for the completion of cases.

•Reducing duplication by using existing reviews or related processes like hospital morbidity and mortality meetings and serious incident reports.

Stakeholders recognised that the above requires administrative input with designated people assigned to coordinating CDOP meetings and a change in information collection.

The main finding was that the aim of the Child Death Review Process was reported as admirable, with great potential to mitigate threats to child health. The process, by which it happens, however, was seen as too labour and time intensive, and produced too few measurable outcomes with limited effect in eliciting change. Stakeholders wanted evidence and feedback of how lessons learnt had translated into a change in policy or what further investigation and research had been initiated as a response. Similarly, the sharing of lessons learnt with other CDOPs was an opportunity that was seldom being used. Other positive aspects noted were an improved bereavement response for parents/carers and families, excellent attendance at rapid response and CDOP meetings, and the enthusiasm and dedication of stakeholders.

## Conclusions

There are a few limitations to this study:

•The findings relate to a CDOP covering two inner-city boroughs with especially diverse and deprived populations and so may not be representative of all CDOPs. However, as the review process is a national requirement, there is some generalisability. Further, the prescriptive nature of the statutory CDOP process suggests that our experience will resonate with other CDOPs. Given the larger number of deaths our panel deals with compared to most, it is likely that our experience is more involved and, potentially, more informative.

•The data collected amounts to only 105 completed cases of the 208 deaths notified, thus limiting the power of the quantitative findings.

•7 of the 25 key stakeholders approached were not available for interview, but the persistence of similar themes from stakeholders suggests a reasonable coverage of views. Not accessing the voice of parents/carers was partly addressed by the bereavement counselors.

This study found that stakeholders were dissatisfied with the current child death review process seeing it as bureaucratic and time consuming partly evidenced by the backlog of cases. Neonatal deaths, especially when extremely premature, presented a tension about whether a full CDOP discussion with all agencies present was necessary or not. Importantly, a need to strengthen the implementation of recommendations and lessons learnt was required.

About 62% of the cases took up to 12 months to complete (compared to 40% in 2010/11 for England) [8].The panel completed discussion of 50.5% of the cases in the 34 month period (compared to 60% for England for 2008/11) [8].This delay was partly attributed to the difficulty in gathering sufficient information on the deaths in a timely fashion. Developing simpler data collection forms is one potential solution with our local area piloting a shorter form C (dubbed the C1) to replace the form B.

Just under half of all deaths reviewed were neonatal deaths with 51.1% of these classified as extremely premature (less than or equal to 28 weeks gestation). The specialist nature of health and social care for children in this age group required recruitment of neonatologists to the CDOP panel in order to adequately assess these cases. Some stakeholders voiced that these cases were still not being appropriately addressed, with too few maternal and social factors considered therefore missing the opportunity to identify public health interventions during pregnancy. Others felt that there was limited value attached to discussion of cases that may simply be non-viable pregnancies. In the US, 14% of the states responding to a survey excluded neonates and extreme prematurity from review, but excluding cases was seen as a weakness as coverage of all deaths is necessary for effective prevention [6]. Clearer guidance on this tension from national leads would be helpful. A separate neonates-only subgroup of CDOP with fewer agencies represented is one potential way forward.

Ensuring that the CDOP recommendations were implemented is crucial, in particular, given that modifiable factors were present in 26.7% of all deaths, higher than the 20% seen nationally [8]. Ways to address the implementation challenges encompass better sharing of lessons learnt between CDOPs and agencies, prompt publications of annual reports with specific, measurable recommendations. Each local CDOP will face its own particular modifiable risk factors. Our inner-city CDOP noted, for example, the management of sickle cell disease and youth-on-youth violence. The involvement of public health professionals is important in helping CDOPs prioritise and identify those interventions which are achievable and of most concern to their area. Also useful could be closer integration of CDOP reports with joint strategic needs assessment so as to inform local Health and Wellbeing Boards (the English Government’s proposed forum for stakeholders to work together to improve health and reduce health inequalities) and their commissioning intentions. This will promote wider partnership ownership and coordinated action across many services. An example is developing a strategy to mitigate youth violence or infant mortality.

The Munro review described the need for a national mechanism for analysing, collating and disseminating local learning [7,10]. Whilst this is important, national expertise should be brought to bear on the emerging trends and themes to identify effective interventions, issue guidance and inform future research priorities. Prematurity and its modifiability is one such example. The US evidence points to the importance of developing a culture within CDOPs to ensure that attention is not overly on analysis and extends to solutions too; and, of having consistency across CDOPs nationally else this may hamper national prevention work [5,6].Munro mentioned a need for a consistent typology and this is echoed by our work as many had difficulty in defining what is “modifiable” and this has persisted despite a national change in terminology on Form Cs from ‘preventable deaths’ to deaths with ‘modifiable factors’ [10].

However, despite this, our stakeholders felt that the process remained worthwhile and was characterised by good multi-agency communication with the potential to further develop into a fundamental tool in improving children’s lives and preventing child deaths. Some potential ways to improve the impact of reviews were suggested and these are in Table 2 below.

**Table 2:** Potential ways that the impact of child death reviews may be enhanced at local and national level

**Policy level**	**Example**
Local area	
Education and training programmes for local workforces	General Practitioner (GP) protected learning time to include updates on e.g. care of the unwell child
Quality of care/commissioning	Setting up incentive payments for services to identify alcohol misuse in families; or, identifying areas for future audits e.g. haemoglobinopathy management
Governance forums and systems	Link learning to serious incident reviews, mortality and morbidity meetings in hospitals
Improving surveillance	Enhanced data collection and sharing in Emergency departments on youth related violence
National or regional level	
Simpler data collection forms	Produce a shortened version of the form B
Guidance on neonates and how best to learn	Advice on the types of neonates that are not likely to need full panel review
National surveillance and review of findings to inform policy	Inform the Healthy Child Programme and other policies e.g. housing

In summary, child death reviews have real potential to reduce deaths. Our panel’s experience is that the current process is bureaucratic, time consuming, should better use related case reviews, deal with neonate deaths more effectively and needs more focus on implementing recommendations.

## Abbreviations

CDOP: Child death overview panel; GP: General practitioner; LSCB: Local safeguarding children’s board.

## Competing interests

The authors declare that they have no competing interests.

## Authors’ contributions

FM analysed the data and interviewed the stakeholders. AM and MW designed, initiated and supervised the study. GH participated in the data interpretation. All contributed to writing the paper. All authors read and approved the final manuscript.
